# Use of telemedicine in the postoperative assessment of proctological patients: a case–control study

**DOI:** 10.1007/s10151-022-02723-9

**Published:** 2022-11-02

**Authors:** F. Gaj, M. Peracchini, D. Passannanti, S. Quaresima, F. Giovanardi, Q. Lai

**Affiliations:** 1 General Surgery and Organ Transplantation Unit, Department of General and Specialistic Surgery, Sapienza University of Rome, AOU Policlinico Umberto I of Rome, Viale del Policlinico 155, 00161 Rome, Italy; 2 Israelite Hospital, Rome, Italy

**Keywords:** WhatsApp, Telemedicine, Outcome, Hemorrhoids, Fistula, Anal fissure

## Abstract

**Background:**

Telemedicine is emerging as an easy way to communicate between patients and surgeons. Use of telemedicine increased during the coronavirus disease 2019 (COVID-19) pandemic. WhatsApp is one of the most common smartphone applications for user-friendly telemedicine. The aim of this study was to evaluate patient perception of health quality and positive outcomes using a diary sent by the patient to the surgeon via WhatsApp during the first post-discharge week after proctologic surgery.

**Methods:**

Ninety-eight patients discharged after proctologic surgery at the Israelite Hospital of Rome and the AOU Policlinico Umberto I of Rome in 1 January–31 December 2019 were divided into two groups: the WhatsApp group (group A), (*n* = 36) and the no WhatsApp group (group B) (*n* = 62). Group A patients received a protocol to follow for the day-by-day diary during the first post-discharge week and sending it by WhatsApp to the surgeon. Group B patients only received recommendations at discharge. The tool's usefulness was assessed by a questionnaire one month after the intervention.

**Results:**

The two groups were homogeneous for age, sex, schooling, employment, and proctologic pathology. Group A patients had less difficulty keeping a diary (*p* < 0.0001). Group A patients had the perception of better follow-up post-discharge (*p* = 0.002). The use of the diary sent by WhatsApp significantly improved the perception of positive post-intervention outcomes (*p* = 0.007). WhatsApp was the only independent predictor of perception of post-surgical positive outcomes (odds ratio = 4.06; 95% CI 1.35–12.24; *p* = 0.01).

**Conclusions:**

The use of WhatsApp in the post-discharge period improves the lifestyle quality of the patients and their perception of the safety and quality of care received.

## Introduction

Telemedicine is the use of electronic information and communication technologies to provide health care at a distance. Information transmitted between doctor and patient can take many forms, including text, audio, radiological images, pictures, and videos [1]. In this setting, mobile technologies are rapidly expanding as a tool for telemedicine [2].

The recent coronavirus disease 2019 (COVID-19) pandemic has driven the medical community to increase the use of strategies to prevent viral transmission [3]. Among them is the migration of health care from hospitals to patients' homes through the strengthening of home care and the implementation of telemedicine [4].

COVID-19 pandemic has led to a revolution in clinical practice due to the drastic reduction elective outpatient treatment and the patients’ reluctance to attend hospital facilities [5].

Currently, one of the most popular nonmedical mobile apps is WhatsApp Messenger. WhatsApp Messenger is a communication tool that allows users to send instant messages, photos, videos, voice messages, and make voice calls over an Internet connection [6].

The possibility of using the app thanks to a mobile Internet connection explains its widespread success [7], and is a user-friendly tool for communication between doctors and patients after discharge [8]. Recent studies have highlighted the possibility of using WhatsApp as a telemedicine tool in specific applications such as oral medicine, orthopedics, and tele-education [9]. However, there is limited experience in the setting of proctology.

We hypothesized that WhatsApp would have a positive effect in the setting of proctology, making it possible to improve patient perception of safety and quality of care received after proctological surgery.

We evaluated this with a post-discharge protocol after proctological surgery based on a day-by-day diary and a direct WhatsApp contact with the surgeon during the first week after discharge. The usefulness of the protocol was assessed using a questionnaire one month after the surgical procedure.

## Materials and methods

From January 1 through December 31, 2019, 98 consecutive patients underwent proctological surgery in the Israelite Hospital of Rome and the AOU Policlinico Umberto I of Rome. The same surgeon (FGa) operated all the patients. From September 1, 2019, 36 consecutive patients received a short post-discharge protocol to complete and send by WhatsApp during the post-discharge period (WhatsApp group, [group A]). This group was compared with a cohort of 62 consecutive patients operated on in January 1–August 31 2019, and retrospectively collected before implementing the protocol (no WhatsApp group, [group B]).

Group B patients received a set of recommendations to follow in the first week after discharge. These recommendations included a series of rules to be followed about hydration, nutrition, use of drugs, and, specifically, pain medications. The patients were not asked to inform the doctor about the daily progress of their recovery.

Group A patients received a specific post-discharge protocol to follow during the first week after discharge. According to the protocol, all the patients collected data in a day-by-day diary covering hydration, food, hygiene, home therapy, evacuation, fever, pain scale, burning scale, and bleeding., ticking the corresponding box. The patients were asked to send the surgeon a photo of the diary on days 3 and 7 using WhatsApp.

The specific characteristics of the protocol, were as follows:

- maintain adequate hydration by drinking 3 L of water per day, indicating the amount of water consumed each day.
- restart usual diet, supplementing it once a day with one of the following foods: prunes, corn, vegetables soup, and cooked vegetables.
- wash the perineal area with water and neutral soap twice a day or after each bowel movement, indicating how many times this was done.
- report the number of evacuations and the stool consistency each day. (Patients were told that it was important to have soft stools every day).
- indicate the intensity of pain and burning sensation daily on a scale from 0 to 10.
- medications: a laxative and lactic ferments were prescribed for daily use, and a mild analgesic was prescribed on demand. A more potent analgesic and a local anesthetic spray were recommended in case of more severe pain.
- report episodes of fever ≥ 38.5 °C and any episode of bleeding during defecation.

One month after surgery, group A patients answered a telephone survey to evaluate the sense of safety and quality of health reported by the patient after the surgery.

Group B patients received the same survey. In this case, the timing from hospital discharge to the administration of the questionnaire was longer.

The questionnaire consisted of six questions:

Question#1 evaluated on a scale from 1 to 4 (insufficient, sufficient, good, excellent) the comprehensibility of the tools given by the physicians at discharge. For group B, the tool corresponded to the list of recommendations, while for group A, it was the protocol with the day-by-day diary.

Question#2 evaluated, with the same scale, the ease of compilation of the post-surgical diary. This question was only for group A patients.

The following questions, to be answered “yes” or “no” directly evaluated the usefulness of the tools given by the physicians at discharge in terms of Q#3) improved outcome of the intervention; Q#4) sensation of being better followed postoperatively; and Q#5) whether the clinical course would have been the same without the post-surgical tools.

Lastly, in Question#6, the patients were asked whether they considered WhatsApp Messenger to be a simple and immediate means of communication about their health with their doctor or whether they considered it a violation of privacy. This question was answered only by group A patients.

### Statistical analysis

Continuous variables were reported as medians and interquartile ranges (IQR). Categorical variables were reported as numbers and percentages. The Mann–Whitney *U* test and Fisher's exact test compared continuous and categorical variables.

A multivariate logistic regression analysis was performed to identify the variables independently correlated with the patient’s perception of an improved post-intervention outcome. Odds ratios (OR) and 95.0% confidence intervals (95% CI) were reported. Variables with a *p* < 0.05 were considered statistically significant. We used the SPSS statistical package version 24.0 (SPSS Inc., Chicago, IL, USA).

## Results

There were 98 patients (53 men and 45 women, median age 54 years [IQR = 46–56]).The characteristics of the investigated population are reported in Table 1.

**Table 1:** Characteristics of the patients in the study

Variables	Group B (*n* = 62)	Group A (*n* = 36)	*P*
Age (years)	51 (43–59)	54 (49–58)	0.2
Male sex	34 (54.8)	19 (52.8)	1.0
Type of surgery			1.0
Abscess	1 (1.6)	1 (2.8)	
Hemorrhoids	30 (48.4)	18 (50.0)	
Fistula	6 (9.7)	4 (11.1)	
Anal polyp	8 (12.9)	4 (11.1)	
Anal fissure	9 (14.5)	5 (13.9)	
Rectocele	8 (12.9)	4 (11.1)	
High education level	46 (74.2)	29 (80.6)	0.6
Employment	45 (72.6)	30 (83.3)	0.3

Group B: No WhatsApp Group; Group A: WhatsApp Group

After comparing the two groups, no differences were observed in age, sex, education level, employment, and proctological pathology.

Group A patients were slightly older (median: 54 vs. 51 years; *p* = 0.2), had a higher level of education (i.e., secondary school or degree) (80.6 vs. 74.2%; *p* = 0.6), and with employment (83.3 vs. 72.6%; *p* = 0.3). However, as already noted, these differences were not statistically significant.

As for the cause of proctological surgery, six different types of procedures were done. In detail, 1 (2.8%) vs. 1 (1.6%) cases of abscess drainage, 18 (50.0%) vs. 30 (48.4%) of hemorrhoids, 4 (11.1%) vs. 6 (9.7%) of fistulas, 4 (11.1%) vs. 8 (12.9%) of anal polyps, 5 (13.9%) vs. 9 (14.5%) of anal fissures, and 4 (11.1%) vs. 8 (12.9%) of rectoceles were reported in groups A and B, respectively, (*p* = 1.0).

The median time from discharge to the questionnaire was 1 month in in group A and longer in group B (5.2 months, IQR = 3.2–7.1).

The results obtained from the answers to the telephone survey are reported in Table 2.

**Table 2:** Responses to the queries of the telephone survey in the two groups

Question number	Group B (*n* = 62)	Group A (*n* = 36)	*P*
	Median (IQR) or *n* (%)		
Q#1: comprehensibility of the tool	3 (2–4)	4 (3–4)	< 0.0001
Q#1: scarce-to-sufficient comprehension	19 (30.6)	1 (2.8)	0.001
Q#2: ease of compilation of post-surgical diary	–	4 (3–4)	–
Q#2: scarce-to-sufficient ease of compilation of the post-surgical diary	–	1 (2.8)	–
Q#3: improved outcome of intervention (YES)	37 (59.7)	31 (86.1)	0.007
Q#4: sensation of being better followed in the postoperative course (YES)	48 (77.4)	36 (100.0)	0.002
Q#5: similar clinical course without the post-surgical tools (YES)	32 (51.6)	15 (41.7)	0.4
Q#6: WhatsApp Messenger useful tool	–	36 (100.0)	–

Group B: No WhatsApp Group; Group A: WhatsApp Group; IQR, interquartile ranges; n, number; Q, question

As for Question#1 (comprehensibility of the tool), the protocol + day-by-day diary was superior when compared with the recommendations alone, with a median score of 4 (IQR = 3–4) vs. 3 (IQR = 2–4) (*p* < 0.0001). In detail, only 1 (2.8%) patient considered the protocol + day-by-day diary as a scarce-to-sufficient tool, while 19 (30.6%) cases in Group B poorly considered the recommendations (*p* = 0.001).

As for Question#2, only 1 (2.8%) patient considered the protocol + day-by-day diary as challenging, and there was an excellent overall median score of 4 (IQR = 3–4).

In Question#3, 31 (86.1%) group A patients considered the protocol + day-by-day diary as able to improve the outcomes of the intervention, while only 37 (59.7%) group B patients had the same consideration for the recommendations (*p* = 0.007).

In Question#4, All group A patients had the perception of being better postoperatively, while only 48 (77.4%) group B patients had the same perception using only the recommendations (*p* = 0.002).

According to Question#5, 15 (41.7%) group A vs. 32 (52.6%) group B patients reported the perception of a similar clinical course without the post-surgical tools (*p* = 0.4).

Lastly, all group A patients reported that WhatsApp Messenger was a useful a tool for communicating with the doctor.

When the variables connected with the patient's perception of post-intervention improved outcomes were investigated, only the WhatsApp tool was an independent factor, with a protective effect (OR = 4.06, 95% CI 1.35–12.24; *p* = 0.01). All the other variables, namely age, sex, level of education, and employment, failed to be significant (Table 3).

**Table 3:** Multivariate logistic regression investigating the variables connected with the perception to improve the outcomes of the intervention

Variables	Beta	SE	Wald	OR	95% CI		*P*
					Lower	Upper	
WhatsApp tool	1.40	0.56	6.20	4.06	1.35	12.24	0.01
Male sex	− 0.55	0.48	1.31	0.58	0.23	1.48	0.25
Age years	0.02	0.03	0.53	1.02	0.70	1.07	0.47
High educational level	− 0.08	0.62	0.02	0.93	0.28	3.10	0.90
Employed	0.06	0.58	0.01	1.06	0.34	3.31	0.92
Constant	− 0.27	1.59	0.03	0.76	–	–	0.86

*SE* standard error, *OR* odds ratio, *CI* confidence interval

## Discussion

In the present study, the WhatsApp tool used for direct day-by-day contact between proctological patients and surgeons during the first week after surgery has shown its usefulness in terms of perception of quality of health and positive postoperative outcomes.

WhatsApp is the most used application for Instant Messaging, and its role in telemedicine has been reported in several studies [1, 3, 10]. In particular, WhatsApp is a recent technology startup founded to build a better short message service alternative [11]. In several regions around the world, particularly in rural areas and low- and middle-income countries, WhatsApp has been shown to facilitate communication among healthcare professionals in terms of faster problem identification and immediate management [11, 12].

The need for healthcare professionals to find alternative methods to follow-up with their patients has dramatically risen due to the COVID-19 pandemic.

This has had a significant influence on the management of patients requiring proctological surgery due to the typically benign nature of their pathologies.

In a study by Campennì et al., telemedicine was adopted to follow-up patients on the waiting list for proctological surgery during the lockdown in Italy. The results showed patients' satisfaction with a telemedicine program [16]. However, some limitations were highlighted, including the inability to perform a physical examination, even if partially possible with video assistance. Uncertainty about data collection and self-reported objective data was found to be another limitation.

Knaus et al. reported their experience of a bowel management program of telemedicine for pediatric patients with congenital or chronic intestinal diseases during the pandemic. Most patients were satisfied, especially because of the lower degree of stress due to fewer trips to the hospital. [17]

A relevant e-consensus on telemedicine in colorectal surgery highlighted how telemedicine represents an important tool, especially in the follow-up, diagnosis, and "decision making" process once the patient has completed the necessary examinations. [18]

A review performed by Colbert et al. underlined some relevant benefits brought by telemedicine to the patients and the healthcare workers, like a reduction in the expenses, no travel costs, less time off work, and potentially lower childcare costs. The risk of communicable diseases is also reduced, as the patient will have limited exposure. [12]

Our results are consistent with other reports, showing the relevance of a user-friendly tool of telemedicine for improving the perception of good health quality. [1–4] Our population was retrospectively evaluated in a pre-COVID period. Therefore, we investigated a "naïve" population in which telemedicine was an alternative rather than a necessity. We wanted to explore a period in which hospital activity was not affected by a severe reduction in services mainly in terms of proctological surgery. Consequently, we tried to minimize the biases potentially caused by selecting patients enrolled during the pandemic.

We found that use of WhatsApp improved patient perception of a positive outcome after surgery, after adjusting the results for potential confounders of this perception, e.g. age, sex, level of instruction, and employment.

When we compared the validity of a postoperative approach based only on general instructions vs. a day-by-day diary to collect patient data, in all cases, the results of the latter approach were superior in terms of perception of quality of care, of safety, and even perception of a positive outcome after surgery.

The necessity to send the results to the surgeon by WhatsApp apparently leads patients follow discharge recommendations carefully, resulting in a feeling of security and high quality of care received.

Our study has some limitations. It is retrospective, and the sample size is small. Nevertheless, we adopted a rigorous statistical approach to avoid potential biases. The sample size limitation was also due to our intention to compare two homogeneous groups of patients receiving or not a telemedicine tool without any bias caused by the COVID-19 pandemic.

Another limitation is that the group B patients answered the phone survey later than the group A patients the patients (median time of 1 vs. 5 months after surgery). This is a critical point of our study, potentially adding some bias to the observed results. Unfortunately, the decision to implement a telemedicine tool was not prospectively implemented with a randomized controlled approach. Only a prospectively designed study would be able to clarify this aspect without any risk of potential bias definitively. Nevertheless, we can assume that a median time of 5 months is not so long to impede a person from remembering a relevant event of his/her life, like some form of discomfort experienced shortly after surgery. Therefore, we are confident that our results should be considered as only slightly inflected by potential biases.

Lastly, we could not clarify if the use of WhatsApp also impacted on the improvement of surgical quality. This impossibility derived from the absence of relevant complications reported in the postoperative course of our series. We can only postulate that direct contact with the surgeon can accelerate his/her intervention in the case of complications.

## Conclusions

Telemedicine is a valuable tool. Using a post-discharge protocol after proctological surgery based on a day-by-day diary and a direct WhatsApp contact with the surgeon gives the patient an easy and efficient way to facilitate communication. Prospective studies on larger populations are required to confirm these results.

## Abbreviations

CI: Confidence intervals
COVID-19: Coronavirus disease-19
IQR: Interquartile ranges
NWAG: No WhatsApp group
OR: Odds ratios
WAG: WhatsApp group
